# Biomineral shell formation under ocean acidification: a shift from order to chaos

**DOI:** 10.1038/srep21076

**Published:** 2016-02-15

**Authors:** Susan C. Fitzer, Peter Chung, Francesco Maccherozzi, Sarnjeet S. Dhesi, Nicholas A. Kamenos, Vernon R. Phoenix, Maggie Cusack

**Affiliations:** 1 School of Geographical and Earth Sciences, University of Glasgow, Glasgow, G12 8QQ, UK; 2 Diamond Light Source, Harwell Science and Innovation Campus, Didcot, Oxfordshire OX11 0DE, UK

## Abstract

Biomineral production in marine organisms employs transient phases of amorphous calcium carbonate (ACC) in the construction of crystalline shells. Increasing seawater *p*CO_2_ leads to ocean acidification (OA) with a reduction in oceanic carbonate concentration which could have a negative impact on shell formation and therefore survival. We demonstrate significant changes in the hydrated and dehydrated forms of ACC in the aragonite and calcite layers of *Mytilus edulis* shells cultured under acidification conditions (1000 μatm *p*CO_2_) compared to present day conditions (380 μatm *p*CO_2_). In OA conditions, *Mytilus edulis* has more ACC at crystalisation sites. Here, we use the high-spatial resolution of synchrotron X-ray Photo Emission Electron Microscopy (XPEEM) combined with X-ray Absorption Spectroscopy (XAS) to investigate the influence of OA on the ACC formation in the shells of adult *Mytilus edulis*. Electron Backscatter Diffraction (EBSD) confirms that OA reduces crystallographic control of shell formation. The results demonstrate that OA induces more ACC formation and less crystallographic control in mussels suggesting that ACC is used as a repair mechanism to combat shell damage under OA. However, the resultant reduced crystallographic control in mussels raises concerns for shell protective function under predation and changing environments.

Marine organisms, such as molluscs^1^ and echinoderms^2^,^3^, use amorphous calcium carbonate (ACC) as a transient phase in the formation of crystalline shell structures, moulding a solid crystalline shell from the inherent disorder of ACC^4^. Phase transitions in biogenic calcium carbonate are therefore important in our understanding of how calcifying organisms produce protective shells. Molluscs and other marine organisms can maintain ACC, even in mature biominerals, as a means of moving high concentrations of insoluble material (CaCO_3_) to crystalisation sites for continued growth and repair^4^. However, even though ACC is a precursor to aragonite and calcite production for shell formation^5^,^6^,^7^, the transformation of ACC into ordered crystalline polymorphs is not well understood. The least stable transient form of ACC is dehydrated^5^ with 1/3 of a water molecule, on average, loosely bound to each CaCO_3_^4^. Under super saturation, this unstable dehydrated form of ACC becomes hydrated with each CaCO_3_ binding to a water molecule. The more stable hydrated ACC can then be transported and deposited by precipitation through dehydration to form crystalline aragonite or calcite for shell formation^4^,^6^. In the larvae of molluscs, *Mercenaria mercenaria* and *Crassostrea gigas*, ACC is an important precursor to aragonite formation in early postset juvenile shells^6^. However, for the sea urchin, *Strongylocentrotus purpuratus*, hydrated ACC is coincident with crystalline calcite^5^ implying that a direct transformation process is unlikely and a complex control for growth and repair is employed^4^.

Increasing carbon dioxide (*p*CO_2_) concentrations leading to ocean acidification (OA) have the potential to alter biomineralisation pathways in molluscs, producing changes in shell structure with implications for the survival of molluscs^8^. Current projections for increases in atmospheric CO_2_ concentration predict a range of 855–1130 μatm by the year 2100^9^ with the potential for a reduction in ocean carbonate saturation states^10^. Increasing seawater *p*CO_2_ can then result in a decrease in the concentration of carbonate impacting intracellular precipitation of amorphous calcium carbonate^1^ with diminished shell ultrastructure^8^. Changes in shell ultrastructure would impact organism control, and mechanisms involved in the shell production.

This study examines the influence of OA on ACC formation in the mussel shell. The common blue mussel *Mytilus edulis* is a globally economically important food resource^11^ that produces a shell comprising two polymorphs of calcium carbonate: aragonite (nacreous layer) and calcite (prismatic layer)^8^. High-resolution X-Ray Photo Emission Electron Microscopy (XPEEM) combined with X-ray Absorption Spectroscopy (XAS) is used to identify mineral phases throughout the *Mytilus edulis* shell structure cultured under current (380 μatm *p*CO_2_) and OA scenarios (1000 μatm *p*CO_2_). The structural phases from the outermost calcite prismatic layer to the innermost aragonite nacreous layer of the shell are determined using Electron Backscatter Diffraction (EBSD). The results demonstrate that OA induces more ACC formation and less crystallographic control in mussels.

## Results

### XPEEM Ca-L edge analysis of the ACC

**Figure 1:**
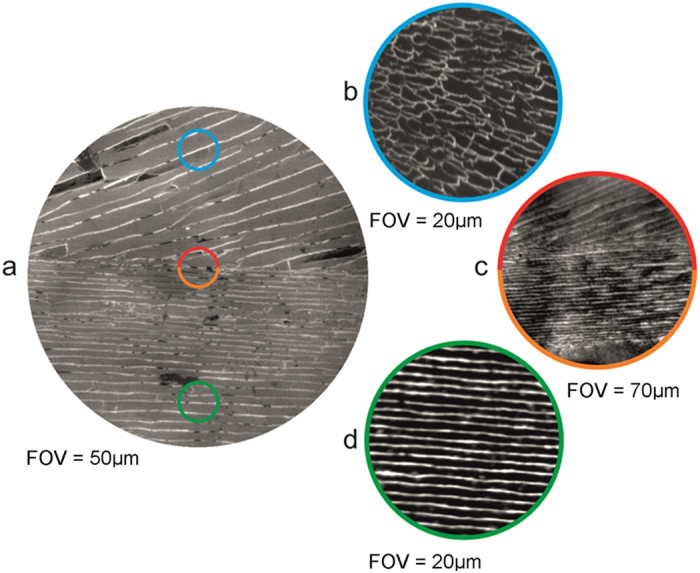
(**a**) Secondary electron emission EBSD image of adult *Mytilus edulis* shell grown under present day conditions (380 µatm *p*CO_2_) showing the calcite (upper half) and aragonite (lower half) and areas analysed. The field of view (FOV) is 50 µm. The coloured circles indicate representative areas where XPEEM images were taken for (**b**) the outer calcite, (**c**) the interface calcite (upper half) and interface aragonite (lower half) and (**d**) inner aragonite. The FOV in each XPEEM image is (**b**) 20 µm, (**c**) 20 µm and (**d**) 20 µm. The image in (**c**) was taken with a higher resolution than the images shown in (**b**) and (**d**).

**Figure 2:**
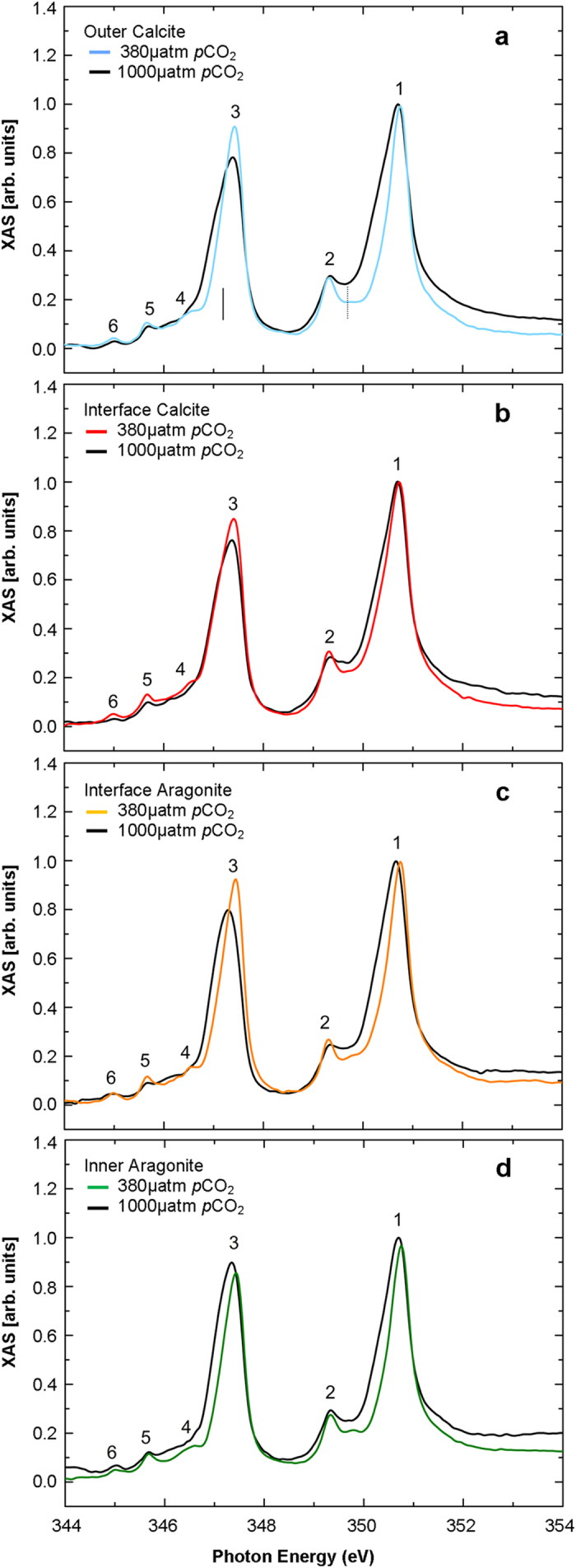
XAS spectra across the Ca L_2,3_-edge of *Mytilus edulis* shell grown under present day conditions for (**a**) the outer calcite, (**b**) the interface calcite, (**c**) the interface aragonite and (**d**) inner aragonite area. The colour of the present day (380 μatm *p*CO_2_) solid line corresponds to the area with the same colour circle in Fig. 1. The spectra for each region of the shell grown under OA (1000 μatm *p*CO_2_) conditions are shown as the solid black line in each panel. The spectra have been overlaid to highlight the increased ACC presence in shells grown under OA in the areas determined to be calcite and aragonite by XPEEM and EBSD. The six features in the XAS spectra are labelled 1–6. The solid vertical line in (**a**) indicates the position of the lower energy shoulder indicative of ACC. The dashed vertical line in (**a**) indicates the position of the peak that increases intensity from the outer calcite to the inner aragonite layer.

There are distinct differences in shell morphology from the outermost to the innermost regions separated by an interface shown in the XPEEM images in Fig. 1. The XPEEM results confirm that the shell structure comprises different polymorphs of CaCO_3_ with the outermost layer comprising prismatic calcite and the innermost area aragonite tablets^12^. Figure 1 shows high-resolution XPEEM images, recorded at the Ca L_3_ edge, for three representative areas of the *Mytilus edulis* shell structure covering the outermost (calcite, near seawater) to the innermost (aragonite, near mussel tissue) region. XPEEM combined with XAS is a powerful element-specific probe of local electronic structure with a spatial resolution of ~30 nm^13^. Figure 2 shows spatially resolved XAS spectra over the Ca L_2,3_ edges from four separate regions of the shell depicted in the XPEEM images (Fig. 1), labelled as outer calcite, interface calcite, interface aragonite and inner aragonite, for mussels grown under present day and OA conditions. In each XAS spectrum at least 6 features are visible and labelled 1–6. These features show a very close resemblance to calculated XAS spectra over the Ca L_2,3_ edges or Ca^2+^ ions in octahedral symmetry^13^,^14^,^15^. We first note that Ca XAS spectra for aragonite regions shown in Fig. 1 are very similar to those reported for synthetic aragonite^16^ except that peak 2 is more intense. However, the spectra for calcite reported in this study do not show the intense feature at the binding energy of peak 5 reported in previous studies^3^,^5^,^16^. The reason for this discrepancy is not clear, but we rule out beam damage since no changes in the spectra were observed with time. The changes in the peak intensities in these spectra are a reliable indicator of the relative abundance of aragonite, calcite and ACC in the shell structures under present day and OA conditions.

### ACC deposition under OA

Comparison of the Ca XAS spectra for aragonite and calcite layers in shells grown under present day (380 μatm *p*CO_2_) and OA (1000 μatm *p*CO_2_) conditions reveals a number of notable changes in the spectra. In mussel shells grown under OA conditions, compared to present day conditions, peak 2 has a marked reduction in intensity. The change in intensity of peak 4 is more difficult to determine, due to the appearance of the low energy shoulder on peak 3, but the analysis reveals a clear reduction in intensity. These changes can be quantified using the ratio of the peak intensities to the background level (Fig. 2, supplementary information Fig. 1). Peak 2 has a ratio ranging from 1.28 to 1.53 and peak 4 has ratio of 0.84 to 0.98 for shells grown under present day conditions. Under OA conditions Peak 2 has a ratio ranging from 0.99 to 1.17 and peak 4 has a ratio from 0.67 to 0.69. A qualitative comparison of the Ca XAS spectra grown under OA conditions in this study (solid black lines in Fig. 2) with those reported for ACC^3^,^5^,^16^ reveals a close similarity to the Ca XAS spectra reported for ACC. A quantitative comparison of the changes in the intensity of peak 2 and peak 4, with the quantitative changes reported^3^, also demonstrates the presence of ACC in the shells grown under OA conditions. Furthermore, peaks 1 and 3 are significantly broader for the spectra recorded from those shells grown under OA conditions in agreement with Rez and Blackwell (2011)^14^ (Fig. 2). The changes in these peak intensities therefore indicate the presence of ACC for those shells grown under OA conditions. Finally, we note that the feature marked with a dotted line at a photon energy of 349.7 eV in Fig. 2(a) gradually increase in intensity from the outer calcite to the inner aragonite indicating a gradual change in Ca coordination across the shell^16^.

### EBSD analysis of crystallographic orientation

**Figure 3:**
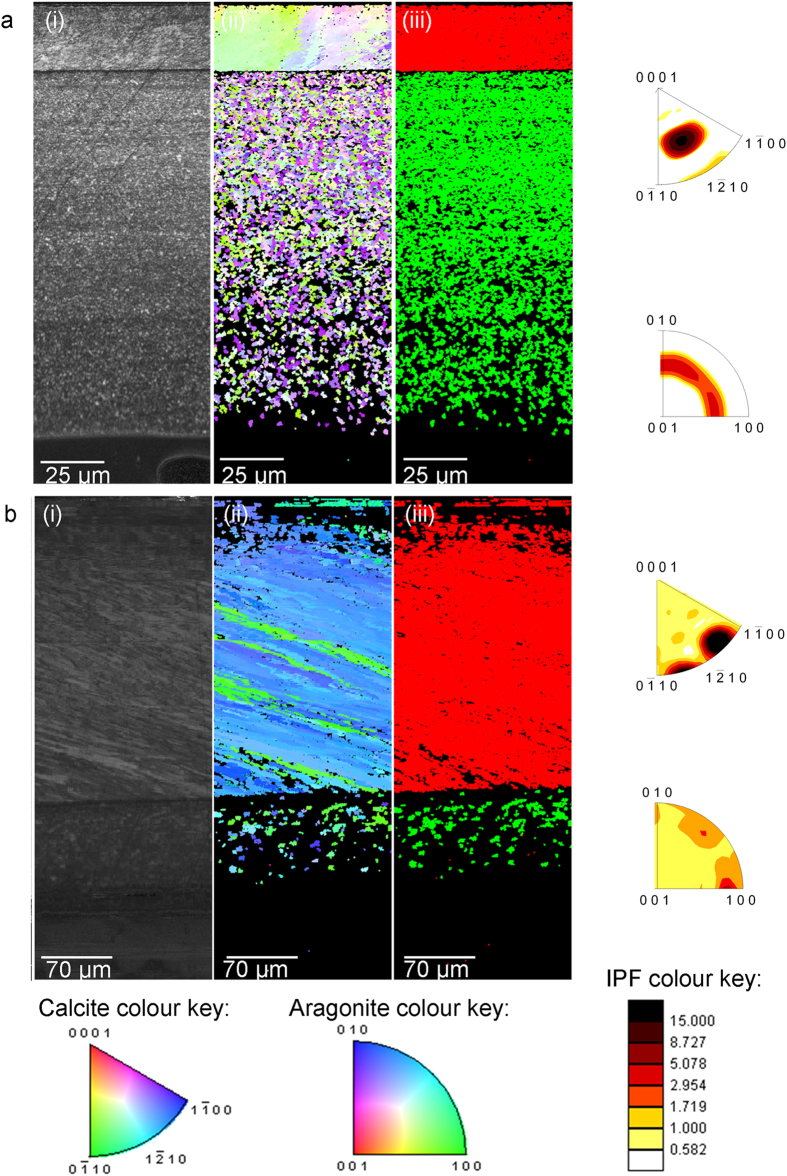
EBSD analysis of mussel shells grown under (**a**) present day (380 μatm *p*CO_2_) and (**b**) OA (1000 μatm *p*CO_2_) conditions. Images present a cross section of the shell from outer calcite (upper area) to the inner aragonite (lower area) for a, (i) diffraction intensity map (DI), (ii) crystallographic orientation map (orientation) according to colour key, [0001] plots are for calcite and [001] plots are for aragonite., (iii) phase map where calcite is shown in red and aragonite in green. Inverse pole figures (IPF) on the right correspond to the crystallographic orientation maps in (ii) using the IPF colour key to indicate strength of CPO or texture. The growth direction of the shells are from left to right in the sections and the IPFs are plotted normal to that view.

The EBSD analyses support the XPEEM results confirming that the shell structure comprises two different polymorphs of CaCO_3_ (Fig. 3). The crystallographic orientation from the outer calcite to the inner aragonite for shells grown under present day (380 μatm *p*CO_2_) and future projected OA conditions (1000 μatm *p*CO_2_) determined using EBSD is shown in Fig. 3. The mussel shells grown under OA have a thinner aragonite layer compared to those mussel shells grown under present day conditions confirmed through phase analysis (Fig. 3iii). Diffraction intensity maps (Fig. 3ai,bi) also indicate that mussel shells grown under OA do not diffract as well as those grown in present day conditions. In addition, crystallographic orientation appears to be less constrained in the calcite areas of those mussel shells grown under OA compared to those shells grown under present day conditions (Fig. 3aiii,biii); represented by increased variation in colour change in the crystallographic orientation maps (Fig. 3bii) corresponding to the colour keys. The reduction in crystallographic control is confirmed by the inverse pole figures (Fig. 3), which indicate the crystallographic preferred orientation (CPO) or spread of data points (orientations) within the crystallographic orientation map (Fig. 3ii). Calcite has a crystallographic orientation angle spread of 40° in those shells cultured under OA compared to 15° in those mussel shells grown under present day conditions (Fig. 3). In contrast, aragonite is more constrained in those mussel shells grown under OA compared to present day conditions, with inverse pole figures indicating a crystallographic orientation spread of 20° compared to 35° respectively (Fig. 3). The diffraction intensity was compared by examining the grey values of the diffraction intensity maps (Fig. 3ai,bi). Aragonite and calcite produced poorer diffraction i.e. higher (darker) grey values of 231.3 ± 31.0 and 228.2 ± 11.6 (Mean ± standard deviation of pixel grey value) in those shells grown under OA compared to 161.7 ± 31.0 and 164.4 ± 28.3 in those shells grown under present day conditions (Supplementary information, Fig. 2).

## Discussion

XAS spectra of the Ca-L_2,3_ edges of a synthetic aragonite and calcite have been compared previously^15^, with notable differences. The XAS spectra reported in this study are the first Ca-L_2,3_ edges for biogenic aragonite and calcite comparison in the mussel *M. edulis*, both appearing as previously reported spectra for synthetic calcite^15^. The similarities in XAS spectra reported for biogenic aragonite and calcite might be due to distortions of the oxygen octahedra in *M. edulis* shells which differ to those presented in other studies or perhaps calcite may not be in an entirely pure phase; this may be expected as organisms exhibit exquisite control over biomineral formation for biological functions^4^,^8^. Nevertheless the changes in the spectra observed for shells grown under present day conditions and shells grown under ocean acidification (OA) conditions are clear in both the aragonite and calcite areas as illustrated in Fig. 3 where calcite and aragonite are readily distinguished by EBSD. Gong *et al*. (2012)^5^ defined three types of Ca L-edge XAS spectra for the sea urchin *Stronylocentrotus pupuratus* spicule: hydrated ACC, dehydrated ACC and crystalline calcite with all three types co-existing within the spicule. As noted, previously reported studies demonstrated that a loss of intensity in peak 2 and 4 along with a small shift in the spectral weight of peak 3 (e.g. marked by the solid vertical line in Fig. 2(a)) indicates the presence of hydrated ACC. Furthermore, a less pronounced loss of intensity in peak 2 indicated the presence of dehydrated ACC^14^,^15^.

ACC is an important transient phase in mollusc shell formation enabling the crystallisation of the shell^1^,^4^. Changes to the availability of ACC within shells grown under OA would impact the ability of the mollusc to produce a protective crystalline shell. Hydrated ACC is present in greater concentrations in mussel shells grown under OA conditions, compared to present day conditions, this is confirmed by XAS spectra indicating a marked reduction in peak 2 and the reduction in peak intensity ratio (<1) for peaks 2 and 4. A comparison with the changes in the reference spectra of 3,15 confirms that the XAS spectra support hydrated ACC presence to a higher degree in shells grown under OA conditions. The presence of more hydrated ACC would imply less crystalline order in shells of *Mytilus edulis* grown under OA conditions. It has been suggested that the structural water presented with hydrated ACC is less important for stabilisation, but more important for lowering the energy barrier for precipitation of ACC^17^. Precipitation of hydrated ACC in those shells grown under OA conditions could then be an energetically cost-effective means of producing ACC^17^ for shell repair^4^. The increases in the availability of hydrated ACC provide an insight into the ability of the mussel to continue shell formation and repair under future OA.

EBSD analyses indicate less constraint over crystallographic orientation of calcite in those mussel shells grown under OA (Fig. 3b) compared to present day conditions, in agreement to previous *Mytilus edulis* studies^8^. Diffraction intensity maps indicate less diffraction of the CaCO_3_ in those mussel shells grown under OA. The higher (darker) grey values represent reduced diffraction intensity in those shells grown under OA. Poorer diffraction in those shells grown under OA would indicate less crystalline structure^12^, which supports the findings of the XPEEM analyses with more ACC present in those shells. The EBSD analyses complement the findings of the XPEEM analyses, which indicate that shells grown under OA conditions contain more hydrated ACC throughout all phases of the shell CaCO_3_ polymorphs.

When cultured under OA conditions, mussels have less control over the crystallographic orientation of their shell components. The results demonstrate that OA induces more ACC formation in mussel shells. The hydrated ACC in the shell may act as a less energetic means of ACC precipitation for shell production or repair^4^. However, the reduced crystallographic control in mussels raises concerns for shell protective function under predation and changing environments.

## Methods

### Mussel collection and culture

Mussels (*M. edulis*) were obtained from the Loch Fyne, Argyll, Scotland (Loch Fyne Oysters Ltd) during October 2012. Mussels were placed into experimental tanks (six L) supplied with natural filtered (1 μm and UV) seawater at Loch Fyne temperatures and ambient (~380 μatm) and OA (1000 μtm) *p*CO_2_^8^. Mussels were fed 10 ml of cultured microalgae (five species of zooplankton, *Nannochloropsis* sp., *Tetraselmis* sp., *Isochrysis* sp., *Pavlova* sp., *Thalassiosira weissflogii* (stock from Reefphtyo, UK,) per tank every other day. *M. edulis* were grown from one year old juveniles for four months of experimental culture, following established protocols^8^.

### Seawater chemistry

Experimental culture was conducted at 380 and 1000 μatm *p*CO_2,_ under seasonal temperatures and day length (light) of the collection site (Loch Fyne, Scotland). CO_2_ was mixed into air lines supplying all experimental tanks, following established protocols^8^,^12^,^18^. Water changes combining seawater and freshwater allowed for maintained experimental conditions similar to those experienced at the collection site in Loch Fyne where freshwater attributes result in existing reduced total alkalinity and carbonate saturation states^8^,^12^. Seawater salinity, temperature, and dissolved oxygen (DO) were checked daily and recorded once a week (YSI Pro2030). Seawater samples were collected (once per month)^8^,^12^,^19^ and total alkalinity (A_T_) was determined using standard semi - automated^8^,^12^,^19^, combining the spectrometric analysis using bromocresol indicator^8^,^12^,^20^, and dissolved inorganic carbon (DIC) using an Automated Infra Red Inorganic Carbon Analyzer (AIRICA, Marianda instruments). Certified seawater references materials for oceanic CO_2_ (Batch 123, Scripps Institution of Oceanography, University of California, San Diego) were used as standards to quantify the error of analysis (Measured TA μmol kg^−1^, 2141 ± 54 μmolkg-1, CRM value 2225.21 ± 0.14 μmolkg-1)^19^. Seawater A_T_, DIC, salinity, temperature and *p*CO_2_ were used to calculate other seawater parameters using CO2Sys^21^ (Supplementary information, Table 1). Seawater samples in triplicate for three areas surrounding the natural Loch Fyne culture have also been analysed for carbonate chemistry (Supplementary information, Table 1)^8^,^12^.

### Shell preparation

Mussel shells were cleaned, dried (60 °C for 48 hours) and embedded in epoxy resin (EpoxyCure, Buehler) blocks. Embedded shells were sliced transversely using a diamond trim saw blade to section the whole length of the shell. New growth was determined through calcein staining of growth bands at the start of experimental culture as detailed in^8^,^22^; any growth prior to this stained growth band was named old growth which occurred prior to experimental culture. The new growth at the outer edge of the shell (containing newest calcite) and towards the newest aragonite formation (containing both newest aragonite and older calcite) was sectioned, and mounted in a resin block before polishing the cut edge of the shell. Resin blocks were ultra-polished using aluminium oxide (0.3 and 1 μm) and colloidal silica (0.6 μm). The sections of ~2 mm were then prepared as thin sections into discs and polished through to 0.06 mm colloidal silica.

### Photo Emission Electron Microscopy

Ultra-polished shell sections of *Mytilus edulis* shell were coated with 1.2 nm platinum (Pt). Sections only from the newest shell growth were examined using XPEEM to ensure grown during 4 months of experimental culture. Shells grown at 380 μatm and 1000 μatm *p*CO_2_ were imaged across the width of the shell from the internal part of the mussel shell containing aragonite to the external calcite. XPEEM images were recorded at the Ca L_3_ edge. A step size of 0.1 eV for energies between 340–344, 0.05 eV for energies between 344–345, and 0.1 eV for energies between 354–355 was used throughout imaging of shell samples to acquire data. Spectra were normalised at the pre-edge and a linear background subtracted, pixels were analysed typically from 5E4 to 2E5 pixels. The spectra shown are from one specimen for each treatment. Multiple areas on the same specimens were analysed to confirm the trends between treatments and two specimens were analysed for each treatment.

### Electron Backscatter Diffraction

Calcite and aragonite crystallographic orientation of the samples was examined using EBSD with a beam voltage of 20 kV under low vacuum mode (~50 Pa) on the FEI Quanta 200 F Environmental SEM with the stage tilted to 70° to examine backscatter Kikuchi patterns^23^. Crystallographic orientation was imaged from the outermost calcite to the innermost aragonite at the centre of the section of shell taken from the new growth of the mussel. Crystallographic orientation maps were produced through OIM Analysis 6.2 software. EBSD results are presented as crystallographic inverse pole figures indicating spread or constraint in crystallographic orientation, the colour key indicates strength of CPO or texture. The orientation maps are also presented with each colour representing a particular crystallographic orientation (confidence index <0.1 removed).

## Additional Information

**Data availability**: The dataset relating to the figures in this manuscript is available at: DOI: 10.5525/gla. researchdata.259

**How to cite this article**: Fitzer, S. C. *et al*. Biomineral shell formation under ocean acidification: a shift from order to chaos. *Sci. Rep.* **6**, 21076; doi: 10.1038/srep21076 (2016).

## Supplementary Material

Supplementary Information

## References

[b1] Huning A. . Impacts of seawater acidification on mantle gene expression patterns of the Baltic Sea blue mussel: implications for shell formation and energy metabolism. Marine Biology 160, 1845–1861 (2013).

[b2] Beniash E., Aizenberg J., Addadi L. & Weiner S. Amorphous calcium carbonate transforms into calcite during sea urchin larval spicule growth. Proceedings of the Royal Society B-Biological Sciences 264, 461–465 (1997).

[b3] Politi Y. . Transformation mechanism of amorphous calcium carbonate into calcite in the sea urchin larval spicule. Proceedings of the National Academy of Sciences of the United States of America 105, 17362–17366 (2008). 18987314 10.1073/pnas.0806604105 PMC2582271

[b4] Addadi L., Raz S. & Weiner S. Taking advantage of disorder: Amorphous calcium carbonate and its roles in biomineralization. Advanced Materials 15, 959–970 (2003).

[b5] Gong Y. U. T. . Phase transitions in biogenic amorphous calcium carbonate. PNAS 109, 6088–6093 (2012). 22492931 10.1073/pnas.1118085109 PMC3341025

[b6] Weiss I. M., Tuross N., Addadi L. & Weiner S. Mollusc larval shell formation: Amorphous calcium carbonate is a precursor phase for aragonite. Journal of Experimental Zoology 293, 478–491 (2002). 12486808 10.1002/jez.90004

[b7] Radha A. V., Forbes T. Z., Killian C. E., Gilbert P. U. P. A. & Navrotsky A. Transformation and crystallization energetics of synthetic and biogenic amorphous calcium carbonate. Proceedings of the National Academy of Sciences of the United States of America 107, 16438–16443 (2010). 20810918 10.1073/pnas.1009959107 PMC2944757

[b8] Fitzer S. C., Phoenix V. R., Cusack M. & Kamenos N. A. Ocean acidification impacts mussel control on biomineralisation. Sci. Rep . 4, 6218 (2014). 25163895 10.1038/srep06218 PMC5385834

[b9] IPPC. Summary for policymakers. in *Climate Change 2007: Impacts, Adaptation and Vulnerability. Contribution of Working Group II to the Fourth Assessment Report of the Intergovernmental Panel on Climate Change* (eds. Parry M., Canziani O., Palutikof J., Van der Linden P. & Hanson C. ) 81–82 (Cambridge, 2007).

[b10] Doney S. C., Fabry V. J., Feely R. A. & Kleypas J. A. Ocean acidification: the other CO_2_ problem. Annual Review of Marine Science 1, 169–192 (2009). 10.1146/annurev.marine.010908.163834 21141034

[b11] FAO. Food and Agriculture Organization of the United Nations Annual report. Fishery and Aquaculture Statistics (2012).

[b12] Fitzer S. C., Cusack M., Phoenix V. R. & Kamenos N. A. Ocean acidification reduces the crystallographic control in juvenile mussel shells. Journal of Structural Biology 188, 39–45 (2014). 25180664 10.1016/j.jsb.2014.08.007

[b13] Krüger P. & Natoli C. R. X-ray absorption spectra at the Ca L_2,3_ edge calculated within multichannel multiple scattering theory. Physical Review B 70, 245120 (2004).

[b14] Rez P. & Blackwell A. Ca L23 spectrum in amorphous and crystalline phases of calcium carbonate. The Journal of Physical Chemistry B 115, 11193–11198 (2011). 21861462 10.1021/jp203057y

[b15] DeVol R. . Oxygen Spectroscopy and Polarization-Dependent Imaging Contrast (PIC)-Mapping of Calcium Carbonate Minerals and Biominerals. Journal of Physical Chemistry B 118, 8449–8457 (2014). 10.1021/jp503700g 24821199

[b16] de Groot F. M. F., Fuggle J. C., Thole B. T. & Sawatsky G. A. L_2,3_ x-ray absroption edgesof d^0^ compounds: K^+^, Ca^2+^,SC^3+^, and Ti^4+^ in O^h^ (octahedral) symmetry. Physical Review B 41, 928–937 (1990). 10.1103/physrevb.41.928 9993787

[b17] Ihli J. . Dehydration and crystallization of amorphous calcium carbonate in solution and in air. Nature Communications 5, 3169 (2014). 10.1038/ncomms4169 PMC4085778 24469266

[b18] Findlay H. S., Kendall M. A., Spicer J. I., Turley C. & Widdicombe S. Novel microcosm system for investigating the effects of elevated carbon dioxide and temperature on intertidal organisms. Aquatic Biology 3, 51–62 (2008).

[b19] Dickson A. G., Sabine C. L. & Christian J. R. *Guide to best practises for ocean CO*_*2*_ *measurements* (2007).

[b20] Yao W. S. & Byrne R. H. Simplified seawater alkalinity analysis: Use of linear array spectrometers. Deep-Sea Research Part I-Oceanographic Research Papers 45, 1383–1392 (1998).

[b21] Riebesell U., Fabry V. J., Hansson L. & Gattuso J.-P. *Guide to best practices for ocean acidification research and data reporting*, (Publications Office of European Union, Luxembourg, 2007).

[b22] Mahe K., Bellamy E., Lartaud F. & de Rafelis M. Calcein and manganese experiments for marking the shell of the common cockle (Cerastoderma edule): tidal rhythm validation of increments formation. Aquatic Living Resources 23, 239–245 (2010).

[b23] Perez-Huerta A. & Cusack M. Optimizing Electron Backscatter Diffraction of Carbonate Biominerals-Resin Type and Carbon Coating. Microscopy and Microanalysis 15, 197–203 (2009). 19460175 10.1017/S1431927609090370

