# Supplementary material for: Biomineral shell formation under ocean acidification: a shift from order to chaos
Source: Sci Rep. 2016 Feb 15;6:21076. doi: 10.1038/srep21076 (PMC4753494; doi:10.1038/srep21076)
Supplement: Supplementary Information [file srep21076-s1.pdf]

Biomineral shell formation under ocean acidification: a shift from order to chaos.

Susan. C. Fitzer[1]*, Peter Chung[1], Francesco Maccherozzi[2], Sarnjeet S. Dhesi[2], Nicholas A. Kamenos[1], Vernon. R. Phoenix[1] and Maggie Cusack[1].

[1] School of Geographical and Earth Sciences, University of Glasgow, Glasgow, G12 8QQ, UK

[2] Diamond Light Source, Harwell Science and Innovation Campus, Didcot, Oxfordshire OX11 0DE, UK

*Tel: +44 (0) 141 330 5442, fax: +44 (0) 141 330 4817, email: susan.fitzer@glasgow.ac.uk.

Peak intensity analysis was performed for peak 2 by dividing the intensity at a photon energy of 349.3eV by the intensity at a photon energy of 349.8eV following methods of Politi et al., (2008) [3]. Peak intensity analysis was performed for peak 4 by dividing the intensity at a photon energy of 346.55eV by the intensity at a photon energy of 346.75eV. The error bars represent the estimated error of the absorption measurement.

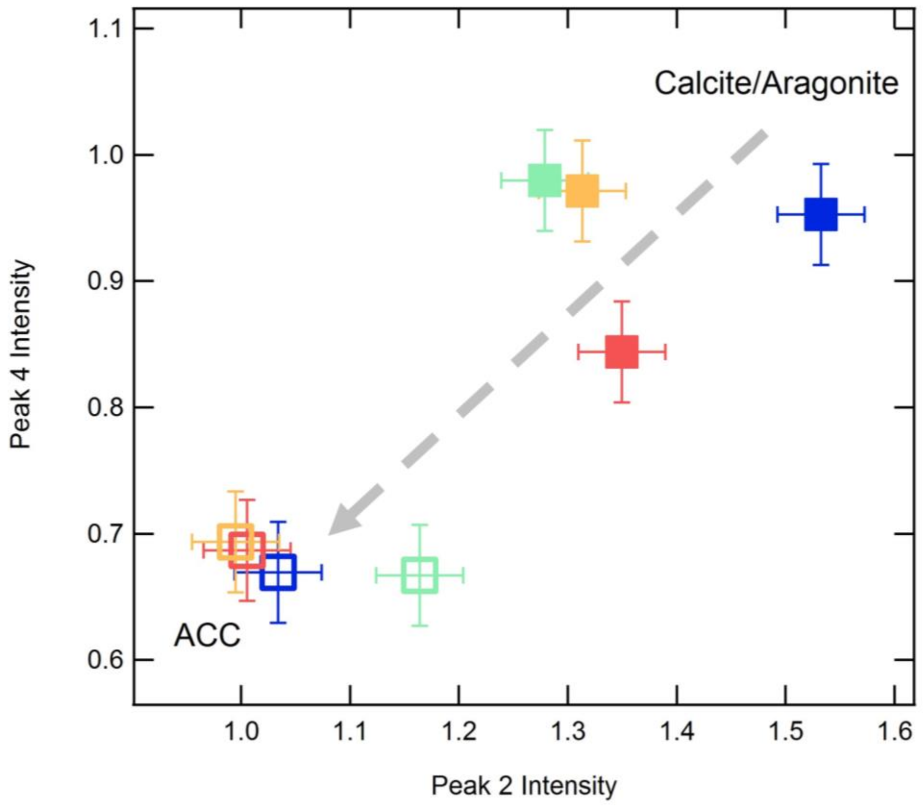

Figure 1. Peak intensity analysis of the Ca L-edge XAS spectra of mussel shells grown under present day (380 µatm pCO_2, solid squares) and OA (1000 µatm pCO_2, open squares)

conditions. Each plot is shown for an image representing a location on a map across the thickness of the shell from outer calcite (blue), interface calcite (red), interface aragonite (orange) and inner aragonite (green). The solid squares represent the centre of mass for the present day samples and the open squares for the OA samples. Arrow represents change to a less crystalline state.

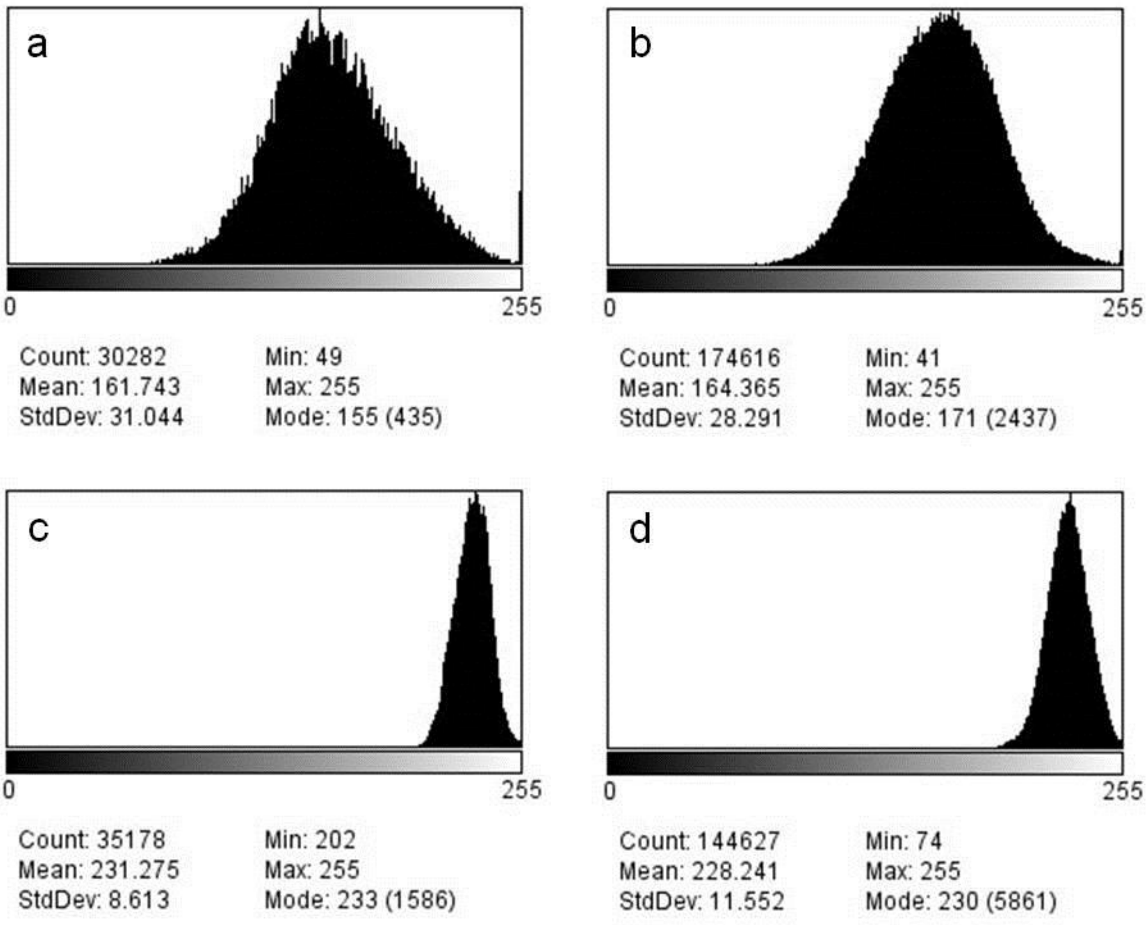

Figure 2. Histogram plot of pixel intensity of the diffraction intensity images using grey value analysis in Image-J software. Diffraction intensity scanning electron microscopy electron backscatter diffraction images were background subtracted (50.0 pixels light back ground) and analysed for grey values of pixel plots for a. aragonite section and b. calcite section of the mussel shells grown in present day conditions and c. aragonite section and d. calcite section of mussel shells grown under ocean acidification conditions. The mean grey value and

standard deviations (StdDev) is presented as sum of the grey values of all the pixels in the selection divided by the number of pixels. A larger grey value represents lower diffraction intensity, as the brighter or white colouration on the diffraction intensity image relates to higher diffraction under electron back scatter diffraction analysis.

Table 1. Experimental seawater chemistry parameters: salinity, dissolved oxygen (DO), pCO_2, total alkalinity (A_T ± standard deviation from the mean). Loch Fyne natural seawater chemistry parameters. Salinity, DO and temperature are averages collected manually throughout experiments, and pCO_2 given is the averaged values logged throughout the six months of experiments (logging every five minutes) using LI-COR ® software. Bicarbonate (HCO_3^-) and carbonate (CO_3^{2-}), calcite saturation state (Ω Ca), and aragonite saturation state (Ω Ar) were calculated from measured parameters using CO_2Sys.

**Experimental condition**	**Salinity (ppt)**	**DO (%)**	**Temperature ($^\circ$C)**	**CO_2 (µatm)**	**A_T (µmolKg^{-1})**	**Ω Ca**	**Ω Ar**	**HCO_3 in (µmol/kg^{-1})**	**CO_3 in (µmol/kg^{-1})**
380 µatm Ambient	33.78 ± 0.74	77.64 ±9.16	8.38 ± 0.29	375.62 ±9.69	722.97 ± 223.38	0.36	0.23	669.4	14.9
380 µatm + 2°C	34.10 ± 2.56	84.21 ±6.62	8.90 ± 0.62	375.62 ±9.69	687.10 ±105.88	0.33	0.21	636.0	13.8
1000 µatm Ambient	38.58 ± 1.27	81.48 ±5.83	9.08 ± 0.46	1132.54 ±31.74	610.28 ±46.96	0.10	0.07	592.6	4.4
1000 µatm + 2°C	35.80 ± 1.71	83.98 ±5.16	9.83 ± 0.63	1132.54 ±31.74	552.76 ±94.08	0.08	0.05	538.2	3.6
Loch Fyne Variability	19.33 ± 7.46	99.36 ±12.99	15.70 ± 4.15	341.17 ±102.57	1261.95 ±416.39	0.88 ±0.47	0.52 ±0.29	1170.56 ±430.42	34.37 ±18.99
Loch Fyne (Lowest total alkalinity values)	17.80	11.6	12.80	250.8 ± 3.7	876.10 ±12.62	0.68 ±0.01	0.39 ±0.01	798.39 ±11.75	29.19 ±0.44
